# Drawing the line between sustainable and unsustainable fish: product differentiation that supports sustainable development through trade measures

**DOI:** 10.1186/s12302-021-00551-6

**Published:** 2021-09-30

**Authors:** Urs Baumgartner, Elisabeth Bürgi Bonanomi

**Affiliations:** 1 Ekolibrium, Hohrainstrasse 5, 3256 Bangerten, Switzerland; 2 Centre for Development and Environment (CDE), University of Bern, Mittelstrasse 43, 3012 Bern, Switzerland

**Keywords:** Fish market regulation, Sustainability practices, Sustainable production, International trade, Ecolabels, Trade policy, Switzerland, European market

## Abstract

**Background:**

Unsustainable production practices and increased demand for fish have aggravated negative social, ecological, and environmental impacts in fisheries and aquaculture. Measures to correct bad practices have mainly been introduced by private actors. However, there is increased demand for state intervention, particularly regarding trade regulations for fish and other agricultural products. Building on discussions about product differentiation through trade measures that favour sustainable products, this study looked at how sustainable and unsustainable fish has been distinguished in Switzerland. In interviewing experts in the fish trade and sales business in Switzerland, the research aimed at understanding the actors and forces that shape the concept of sustainable fish in the country.

**Results:**

Three ways of product differentiation for sustainable fish by private actors were identified in Switzerland: ecolabels, “Swiss produce”, and recommendations in the form of a “consumer guide for fish”. Currently, price is the main constraint on consumption of sustainable products in the country. Defining “sustainable fish” is challenging and subject to interpretation. All existing measures to differentiate sustainable from unsustainable fish products in Switzerland have shortcomings, particularly in terms of discrimination and inclusiveness. Fish ecolabels play a key role in product differentiation, but experts believe that they fail to accommodate all aspects of sustainability.

**Conclusion:**

Our findings imply that the Swiss state should play a more important role if it aims to fulfil the promise of article 104a of the Swiss Constitution, which seeks to foster sustainable production and cross-border trade relations that contribute towards this goal. Preferred trade treatment for sustainable fish products is a potential option to increase the production and consumption of sustainable fish. When designing measures for product differentiation, a careful choice is paramount to address sustainability in a holistic, inclusive, and transparent way and in order not to violate existing trade obligations. Due to similarities between the Swiss and other fish markets, we assume that governments in general and members of the European Union in particular must play an active role in shaping the definition and trade of sustainable fish products if they seek to comply with their sustainability commitments.

**Supplementary Information:**

The online version contains supplementary material available at 10.1186/s12302-021-00551-6.

## Introduction

A growing population and increased demand for fish^1^ have aggravated issues around overfishing and unsustainable use of aquatic resources worldwide and have consequently stimulated discussions on how to meet demand for fish produced in an environmentally and socially sustainable way [37]. In response to pressure from conservation nongovernmental organisations (NGOs) and consumer organisations, various efforts have been made to improve production practices and value chain performance along fish value chains. Most prominent are private efforts such as eco-certification, direct sourcing schemes, and consumer advice in the form of “seafood guides” [29]. However, the benefits and impacts of efforts by private actors on fish sustainability have been questioned on different fronts [2, 15, 17, 30]. Looking forward, Roheim et al. [29] suggest that stronger action by states might improve prospects for a sustainable fish market in the future. From a compliance perspective, limiting the production and consumption of fish that have unnecessarily negative social, ecological, and environmental costs could be a mandatory requirement for states to meet their international commitments, such as the Sustainable Development Goals 2030 or the Aichi Biodiversity Targets.^2^ Furthermore, a stronger role would allow states to react to demands from their citizens, who clearly seem to prefer fish from sustainable sources over other alternatives [5, 11, 20].

The idea that the public sector should play a more important role is taken up by Bürgi Bonanomi and Musselli [8], who propose that states ought to interfere in global value chains with a stronger focus on sustainable development. Analysing trade agreements, the authors observed that whereas trade terms often affect the agriculture sectors of partner countries, current trade agreements do not generally include provisions that seek to mitigate the negative impacts of production practices on vulnerable groups or the environment. To overcome this weakness, Bürgi Bonanomi and Musselli [8] suggest that future trade agreements should be more inclusive and have a stronger focus on potential impacts rather than on decreasing tariffs only. As a specific example, the authors mention product differentiation that could be applied with sustainable development in mind, an intervention that so far has received the most attention in public procurement.^3^ Similar to the EU’s approach to combat illegal, unreported, and unregulated (IUU) fishing,^4^ states could influence production practices in the partner country by providing preferential trade terms for sustainable products compared with likewise products that are produced at high external costs [9].

The power of such an intervention can be illustrated in the example of fish consumption in Switzerland. Like many European countries, Switzerland depends heavily on imports to meet its domestic demand for fish. For the past two decades, imports have made up over 96% of the country’s total fish consumption, reaching a high of 97.8% in 2017 [7]. Likewise, consumer demand for sustainably produced food products is also high [22]. What is more, consumer preference for sustainable agriculture production and trade has a high priority in national agricultural policy: to guarantee the supply of food for the Swiss population, article 104a of the Swiss Constitution seeks to strike a balance between opening markets for agriculture imports and safeguarding local production. More specifically, it demands, among others, creating conditions for “cross-border trade relations that contribute to the sustainable development of the agriculture and food sector”. However, Swiss imports of fish are not currently screened for sustainability, and efforts to mitigate the negative effects of fish consumption are subject to voluntary action by private actors. Given Switzerland’s high dependency on imports of fish products as well as the rigorous demand for sustainable agricultural production, it could be argued that the government must use trade policy to interfere in fish value chains.

The question is, how can the state do so without challenging agreements with already established institutions such as World Trade Organization (WTO), as has happened in three broadly discussed cases involving fish products?^5^ If the Swiss government applied different tariffs for sustainable and unsustainable fish products to foster sustainable development, how would it draw the line between what is sustainable and what is not? As Roheim [28] and Parkes et al. [24, 25] highlight, it is highly complex to define sustainability in the context of fish value chains. Given this complexity, how could the Swiss government translate such a concept into practice? Could it build on private efforts with the same objective, as it has done in a recent trade agreement despite substantial critique?^6^ To the authors’ knowledge, design of differentiation in fish products addressing sustainability criteria and considering trade law has not yet been explicitly addressed in academic literature.

With the goal of closing this gap, this study therefore focuses on understanding how sustainable and unsustainable fish can be differentiated and how to incorporate such distinction into trade policy.

## Methodology

To answer the research questions, we followed a case study design [6, 41]. Taking the Swiss fish market as our unit of analysis, we looked at how sustainable fish has been defined, what measures the private sector uses to foster the consumption of sustainable fish, and which key elements are considered for fish product differentiation to meet sustainability criteria. In addition, we analysed how current means to differentiate sustainable from unsustainable fish products compare against certain criteria that must be followed, such as not to violate the principles of non-discrimination and proportionality under WTO law.

Following Yin [41] we used multiple techniques to collect data. First, we interviewed 13 experts familiar with fish trade in Switzerland. From February to December 2020 these experts were interviewed by Skype, phone, and face-to-face using semi-structured interviews. Interviews were carried out in German, French, or English using a German questionnaire as guidance (see “Additional file 1, 2, 3, and 4” for details). Interview length and subject areas covered depended on the background and availability of interviewees and stretched from 1 h to as many as several hours for those that required repeat sessions. Interview questions were targeted at understanding the sustainable fish movement in Switzerland, including actors, objectives, definitions, means, and impacts. Questions also aimed at gaining an overview of the interviewees’ perceptions of eco-standards and consumer labels and their experiences with fish labels as a tool for product differentiation.

After first evaluating the findings, we then carried out an online survey using Microsoft Forms. The main reasons for this were to reach more interviewees, despite the emerging communication challenges from the coronavirus pandemic, and the limited availability of interviewees. The online survey included 20 largely open questions, for which respondents took an average of 35 min to reply.^7^ Out of 42 experts invited to participate in the survey, 23 accepted (4 of whom had already participated in the earlier interviews).

Data from the interviews were complemented with an online search. This involved inquiries for specific data, such as trade information or market shares, and consultation of specific sites, such as consumer guides for sustainable fish and websites of key actors. In addition, we also carried out a search by topic using the terms “sustainable fish Switzerland”, “Swiss fish”, and “Swiss aquaculture” in German, French, Italian, and English.^8^ The objective was to reach a broad coverage of our unit of assessment. This search was random rather than structured and targeted “completeness” of data.

We then synthesised and analysed data from the interviews and online surveys using “thematic analysis” [6]. The guiding “themes” included aspects of sustainability [27], key criteria to WTO law according to Bürgi Bonanomi and Tribaldos [9]:

i. inclusiveness (to cover a multitude of production methods without imposing specific cultural values),
ii. non-discrimination (domestic producers must be benchmarked against the same sustainability criteria as producers in the importing countries),
iii. efficiency (the intervention should be as minimal as necessary),
iv. effectiveness (measures should effectively facilitate a sustainable transformation),

and themes that emerged during the interviews, such as “Swiss fish”.

We then triangulated findings with data from the online search. Identified data gaps were complemented through additional interviews with interviewees from the first group of interviews and by further online searches.

Interviewees and survey participants consisted of experts working at different management levels who had a mix of insights into fish value chains. Key selection criteria were a sound understanding of the Swiss fish market and a good knowledge of the sustainability agenda. Interviewees represented a wide range of business sectors and working areas (Table 1). They were selected using the author’s personal contacts, a snowball system (asking interviewed experts for other suitable contacts), and an online search (using platforms such as LinkedIn, Xing, and company websites of relevant organisations).

**Table 1:** Number of interviewees according to business sector (left) and working area (right)

Business sector	Number	Working area	Number
Producer	2	Trade, sales	12
Retail	12	Sustainability	6
RFS	3	Quality management	2
Trade	7	Marketing	5
Label organisation	3	Research, consulting	4
NGO, academia, other	5	Policy, governance	3
Total	32	Total	32

### Calculating the share of “sustainable fish” products in the total Swiss fish market

The share of sustainable products in the Swiss fish market (Fig. 2) was calculated using information from the World Wide Fund (WWF) about the sustainability performance of its retail partners,^9^ confidential data about fish market shares in Switzerland shared by two interviewees, feedback from interviewees, company websites, and public data. We used triangulation to estimate the best possible figure that we had verified, again using interviewees for confirmation. For a distinction between sustainable and unsustainable products, we used the “WWF approach”, deeming as sustainable all products with a WWF score of 1 or 2, including the labels “MSC” (Marine Stewardship Council), “ASC” Aquaculture Stewardship Council) and “organic” regardless of potential flaws. Taking a holistic perspective to sustainability, the shares of “sustainable fish” in the overall fish market would thus likely be smaller.

### Findings

#### Sustainable fish is a question of place and interpretation

Overall, the importance and level of sustainability of fish products in Switzerland vary mainly across distribution channels and, to a lesser degree, on other factors, including cultural aspects.

While opinions differ on the share of sustainable products in the Swiss retail sector, respondents point to the obligation of reducing the share of unsustainable products that the main retailers have due to internal and external commitments.^10^ However, there is a consensus that within the restaurant and food service (RFS)^11^ sector, sustainability is far from playing an important role. High competition and price-sensitivity seem to hinder stronger acceptance, and price differences were flagged as the main reason why not all fish products were sustainable in the retail sector. According to interviewees, price differences between sustainable and unsustainable products depend on the species and can be large for certain species. In addition, the sustainability of products is much more challenging to communicate within the RFS sector than the retail sector. Unlike with packaged products, “fish labels can hardly be attached to a plate”, as one respondent put it. This limits the visibility of ecolabels in restaurants. Representatives from the sector further explained that availability in RFS was a key requirement and mostly translates to species and freshness, coupled with a demand for small quantities of fish only. This necessitates remarkably high flexibility for the seller and makes it all the more difficult to put sustainability of products at the centre. Others explained that in gourmet and speciality shops, availability was paramount in terms of a broad product range. In such shops, rare or exotic products might be a key sales point. In terms of sustainability, however, these products often score poorly. In contrast, retail shops focus more on species that are consumed “in masses”, including salmon, shrimp, tuna, and cod. Experts pointed out that particularly for discounters, which only feature a few fish items, selling uniquely sustainable fish was not much of a challenge compared with a restaurant or gourmet shop, where guests look for an alternative to the fish they already eat at home. For these reasons, experts flagged the RFS sector as a big challenge for sustainability.

Some respondents also explained that cultural or geographic factors influenced the level of sustainability. For example, consumers of Mediterranean origin had specific preferences due to their cultural heritage. These consumers appreciate fish with certain cultural value regardless of their sustainability performance.

The level of sustainability is also a question of interpretation. While major retailers and other key actors use the “World Wide Fund (WWF) seafood guides”^12^ as an orientation for sustainability, there is no agreement of where to draw the line between sustainable and unsustainable products. The WWF seafood guides, which are meant to help consumers make the right buying decision, separate fish products into four categories: green for “recommended”, orange for “acceptable”, red for “avoid”, and blue for “recommended labels”.^13^ WWF-recommended labels include “ASC”, “MSC” and “organic”. According to the WWF, “acceptable” products are not sustainable. On pressure from members of the Seafood Group,^14^ however, WWF Switzerland had allowed members to advertise such products as sustainable in the past, an exception that ended on December 31, 2020. Starting in January 2021, WWF recommendations in Switzerland were aligned with communication from other WWF offices, and certain fish that could be sold as “sustainable fish” in 2020 are no longer considered sustainable in 2021, even though production practices are exactly the same as before.

#### Swiss or “local” produce as a sustainable option

Independent of WWF recommendations, there appears to be a common perception that domestic fish is sustainable. Many retailers feature products labelled as “regional produce”. While suggesting a “preferred product” compared with others, these label schemes do not integrate any environmental objectives. Migros’ “*Aus der Region*” label simply defines the geographic scope of production. Coop does not publish information on what a producer must fulfil for its products to be sold under the label “*Miini Region*”, though the name suggests similar requirements. Nevertheless, most interviewees shared the view that Swiss produce is sustainable. Among others, experts believe that “Switzerland disposes of sound regulations” and “compared to imported fish, the eco-footprint of Swiss fish is lower”. They further observed that Swiss fish would in any case be better than imported fish, mainly for two reasons. First, in the words of one respondent “there were no reports of disturbing production practices, such as those exposed in the pangasius (*Pangasionodon hypophthalmus*) industries in Vietnam”. The second reason is found in short value chains with reduced energy use for transportation. According to respondents, short transportation routes result in positive environmental outcomes compared with imported fish products, which require long transportation routes by ship and road or (in the worst case) even by plane.

Others were more sceptical and believe that “Swissness” was pure marketing, a claim supported by the observation that availability of local products was more important than their sustainability performance. For example, some lake fish species have a strong local tradition in Switzerland, such as rainbow trout (*Oncorhynchus mykiss*), pike-perch (*Sander lucioperca*), and lake whitefish (various *Coregonus* spp., locally labelled “Felchen”). These are apparently a big challenge for the entire Swiss fish sector due to reduced wild populations and decreasing catch volumes in recent decades. According to respondents, however, imports cannot bridge the production gap of traditional lake species, even if their sustainability performance were better compared with local produce, simply because they are not Swiss produce.

Other interviewees expressed particular concern regarding adequate legal requirements for aquaculture production in Switzerland. There is no national aquaculture framework, and regulations therefore strongly vary throughout the country. Furthermore, small-scale fish farming in traditional cattle or pig farms as an additional source of income is largely exempt from regulations that apply to commercial fish farms. In contrast to professional fish farms, agriculture farmers are not required to have fish knowledge, and effluent water does not need to be treated before discharge. In addition, there are limited requirements regarding animal welfare and chemical use. Those experts familiar with fish production and legal requirements in Switzerland therefore view the proliferation of “amateur” fish production as damaging.

#### WWF’s influence on the definition of sustainable fish

**Fig. 1:**
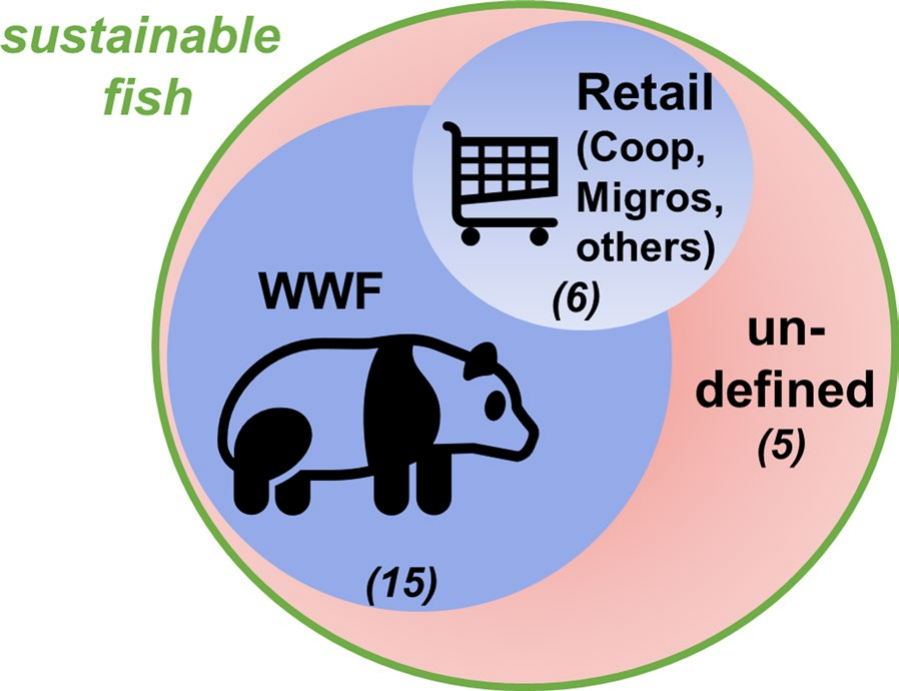
Actors that shape the definition of “sustainable fish” in Switzerland. Number in (italic) at the bottom of each circle shows the number of respondents that indicated the respective actors (*n* = 25, multiple choices possible)

The influence of the WWF on the sustainable fish movement in Switzerland is undisputed among experts. Out of 25 experts that answered the question, 15 believe that the WWF is the organisation that most influences the definition of sustainable fish. The other big driving force, mentioned by six respondents, is the two major retailers Coop and Migros, as well as the retail business in general, which in turn are influenced by the WWF (Fig. 1). Five experts pointed out that the term sustainable fish is not officially defined by anyone and that, consequently, different actors use varying definitions.

The WWF’s theory of change lies in transforming value chains by working at the consumer end with two audiences. Consumers are the main target, since a change of consumption depends on the choices they make. To this end, the WWF has developed its consumer guides for fish to help consumers make informed choices when buying fish. Its recommendations are based on assessments using two methodologies (fisheries versus aquaculture), which the WWF developed together with partner organisations^15^ to assess the sustainability of fish production units. Not obvious for consumers (who only see the WWF’s colour scheme) is the “score” that results from these assessments. Every analysed production unit receives a score between 1 and 5, where 1 is the best and 5 the worst. Products with a score of 1 or 2 are considered sustainable fish, whereas those scoring 3, 4, or 5 indicate unsustainable production practices, even though, as mentioned already, in Switzerland a score of 3 could be marketed as sustainable as recently as the end of December 2020.

Seven experts (*n* = 25) mentioned either the WWF’s seafood guides or the WWF score^16^ as the dominant mean for implementing sustainability targets. Together with 14 positive replies for “labels”, the guides and scoring system are believed to be key in driving a sustainable transformation in the Swiss fish sector.

The second main target of the WWF is key retail companies, with whom it has entered into “partnerships” that aim to improve their product ranges together. To have a sector-wide impact, the Seafood Group was created in 2009, consisting of representatives from the retail and RFS sectors and coordinated by the WWF through a roundtable. The group was active until the end of 2015, when it was apparently dissolved due to “different agendas” and lack of capacities to fulfil the group’s vision. While representatives from the RFS sector are no longer in dialogue with the WWF, the organisation continues to work with the biggest retailers, such as Coop, Migros, Denner, and Lidl, as well as Bell and Micarna, two subsidiaries of Coop and Migros, respectively, with which they maintain bilateral partnerships.

A minority (4 out of 25) of experts was critical towards the power of the WWF and its business partnerships with major retailers, as they see these as monopolising the sustainable fish paradigm. Judging from their overall feedback, it could be observed that some of these rather sceptical experts were nevertheless—and apparently involuntarily—influenced by the WWF’s interpretations of sustainable fish. They mentioned that the WWF-recommended labels “ASC”, “MSC”, and “organic” as well as the WWF score are, when taken together, a good definition for sustainable fish.

**Fig. 2:**
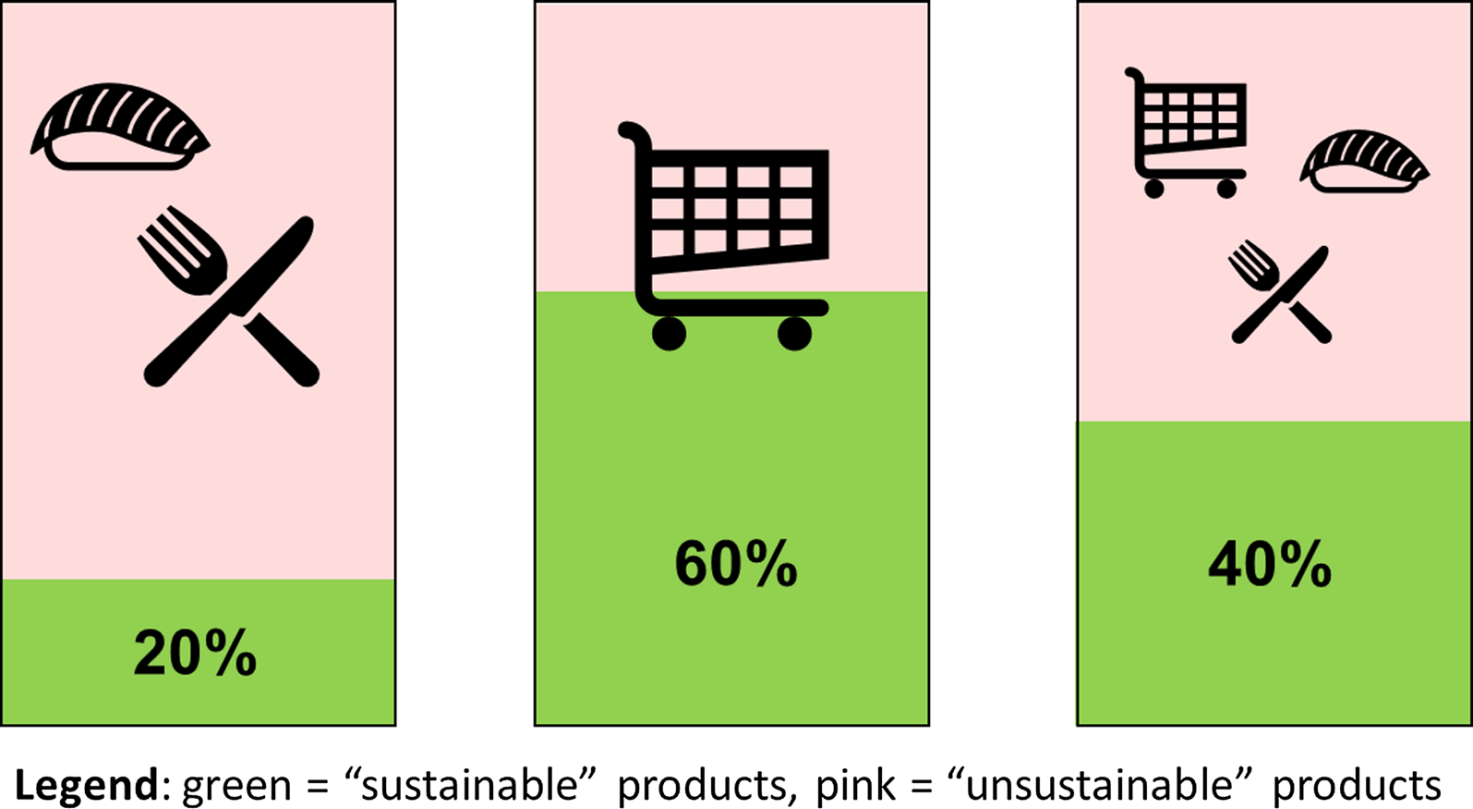
Estimated share of “sustainable fish” products in Switzerland. RFS sector (left), retail market (middle), and total fish market (right). For the details of calculation please refer to the ‘Methodology’ section

Using this approach, an estimated 40% of all fish consumed in Switzerland can be deemed sustainable,^17^ with the share of sustainable products being much higher in the retail sector compared to RFS (Fig. 2).

#### The challenge of defining “sustainable fish”

**Fig. 3:**
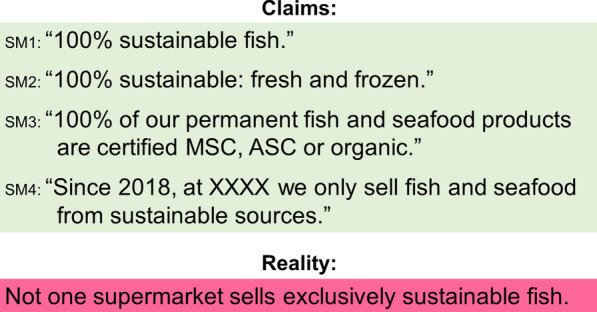
Fish sustainability claims of four major supermarkets compared with the reality. Translated from original language by author. See “Additonal file 5” for references

Overall, we observed that experts had difficulties defining sustainable fish in their own words. Many definitions focused on maintaining fish biomass without considering other dimensions of sustainability,^18^ even where interviewees criticised the absence of a holistic perspective as a weakness in existing rating schemes. Interview feedback indicates that the challenge to clearly draw a line between sustainable and unsustainable fish was one of the biggest hindrances in achieving a better performance of the sector. Respondents explained that because the definition of sustainability is subject to individual interpretation, many actors simply use the concept most useful for them, with the goal being to claim that they produce, trade, or sell sustainable fish, even where this is not true. This observation is underlined by the fact that four out of the six retail companies with the highest fish sales (the four sharing an estimated 85% of total market share in the retail sector) all claim to exclusively sell sustainable fish, whereas in reality none does (Fig. 3).

Two respondents fear that it might be impossible to fulfil all criteria that make a fish product sustainable, since, as one explained, “there are always trade-offs between different aspects of sustainability”. The other illustrated their point by referring to a controversy they repeatedly found themselves in. When switching to a labelled product from unlabelled fish, they had to change their former supply from the Mediterranean Sea to a fishery farther away. For fish to be sold fresh, this generally means changing the means of transportation from truck to plane, a practice the respondent considered totally irresponsible from a sustainability perspective, though the fish products are then considered sustainable.

#### The influence of fish labels

According to a majority (16 of 25) of respondents, labels play a key role in consumer perception about fish. The WWF has selected specific ecolabels for products that it considers unconditionally sustainable—regardless of the outcomes when applying its assessment methodology. These labels include MSC for fisheries and ASC and organic labels for farmed products. The share of labelled fish in overall sales according to one of the WWF’s recommended labels is a key performance criterion of the organisation’s partners. As a result, the continuous increase of labelled fish as a percentage of fish sales is one of the main sustainability targets. This approach was criticised by eight interviewees who believe that it leads to a proliferation of big corporations at the expense of smallholders, thus contributing to further monopolisation of the sustainability paradigm.

Ten experts (*n* = 25) pointed out that in addition to the “WWF-imposed labels”, other fish labels can also be considered sustainable. Friends of the Sea (FOS) was mentioned five times, GlobalGAP three times, Fair Trade USA (FTUSA) twice, and Best Aquaculture Practice (BAP) and AquaGAP once each. Two experts believed that GlobalGAP was particularly relevant from a sustainability perspective. The standard has existed much longer than the ASC, is open to more fish species, and has been available for many species before the ASC standard. The experts also believe that overall differences between the ASC and GlobalGAP were minimal, with GlobalGAP being even stricter in certain key areas of concern, such as feed inputs. The similarity could best be illustrated by the large number of ASC certified farms that are also certified according to the GlobalGAP standard. The two respondents therefore regret that the WWF does not acknowledge GlobalGAP as equivalent to the ASC and believe that market interests might influence the decision more than sustainability objectives.

**Fig. 4:**
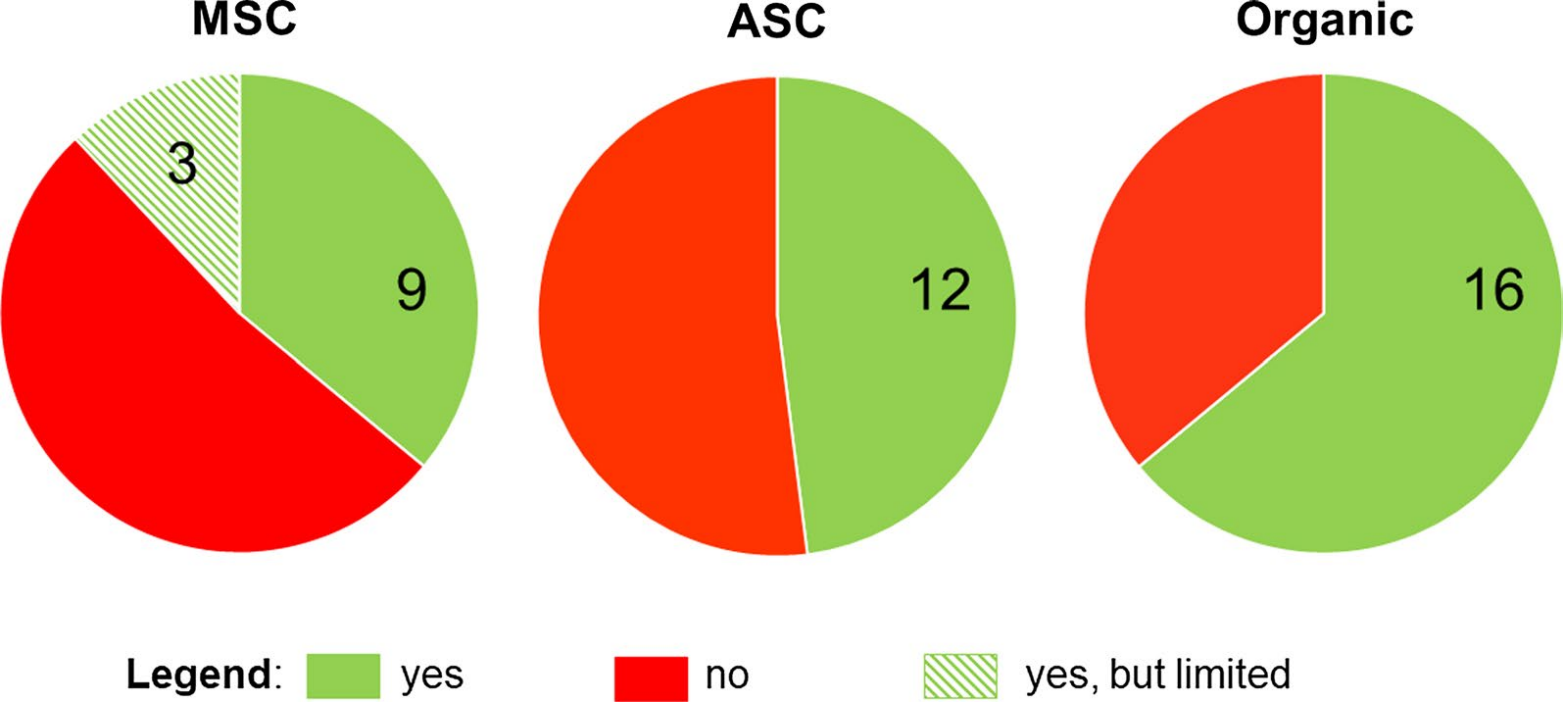
Share of experts (green) who consider WWF-recommended ecolabels as representing a “sustainable choice”. Numbers show affirmative responses (*n* = 25)

Four interviewees questioned the WWF’s practice of promoting all MSC-certified products as sustainable, despite the WWF having logged objections to several MSC certifications because it believes the corresponding fisheries are unsustainable. Twelve of the 25 interviewed experts think that MSC-labelled fish can be considered sustainable, though three had reservations regarding the sustainability of this standard (Fig. 4). The ASC label received the same number of approvals, while 16 experts approved of organic labels.

Respondents explained that labels play a particularly important role in the retail sector, as they make simple tools to communicate sustainability to consumers. As a result, consumer-facing labels such as “ASC”, “MSC”, and “organic”, which enjoy high recognition among consumers, are preferred over sustainability standards that are not clearly communicated to consumers, such as GlobalGAP. Lack of visibility was thus mentioned as a drawback for lesser-known standards.

Regardless of their use, experts expressed concerns regarding ecolabels. A key concern that was repeatedly brought up is the labels’ narrow definition of sustainability. Respondents explained that the MSC and ASC only focus on environmental sustainability but fail to include social and financial considerations. Other experts resent a better integration of social impacts, economic concerns, and animal welfare. Difficulties for smallholders or smaller fisheries to achieve certification was another concern regarding labels. In addition, conflicts of interests (e.g. economic targets as the main goal above sustainability) and inadequate sustainability criteria within these are further constraints of fish labels, though they were cited less frequently. Constraints include the practice of labelling farmed fish as sustainable, even where the feed required to raise the fish contained more food grade fish than the production system produced as net output (fish-in-fish-out ratio > 1). In the respondents’ view, only a production system with a positive net gain should be considered sustainable. They clarified that, from this perspective, carnivorous species such as salmon, seabream, yellowtail and cobia were impossible to farm sustainably, even if their availability under the ASC suggested otherwise. Further constraints include the perception that, according to the respondents, certain catch methods, such as bottom trawling, could never be sustainable due to habitat destruction but are nevertheless heavily represented in the MSC label.

#### Lack of transparency, social criteria, and better integration of animal welfare

Experts identified lack of transparency, lack of social criteria, and better integration of animal welfare as the main flaws in current sustainability agendas for fish. Regarding transparency, respondents observed that while the WWF stressed transparency in value chains as a key to sustainability, the biggest weakness of its fish guides was a lack of transparency. The WWF assigns scores to different value chains without providing assessment details, justification, or explanations. Interviewees also highlighted that current legal requirements fall short to foster transparency. A mentioned example is mandatory product declarations for fish that are restricted to a capture area and capture method category. To determine the sustainability of a product, more detailed information is required, such as the specific catch method. Since this information was not officially required, it cannot be verified. As one interviewee put it, “fish traders can manipulate information to their advantage”, a claim supported by others.

Interviewees further pointed to inconsistencies in ecolabel schemes. Three respondents claim to know that traded volumes of specific MSC and ASC products exceed the capacities of the corresponding production units, which they interpret as proof of systematic cheating. The failure to better integrate social criteria in “sustainable fish” definitions is a further critique from interviewees. Three respondents regret that fish welfare has so far not had a major role in defining sustainable fish.

## Discussion

From the three established means to differentiate between sustainable and unsustainable fish products in Switzerland, all appear to have shortcomings. A case in point is Swiss or local produce, which receives high support regardless of objective sustainability criteria. A reason for experts to predominantly perceive Swiss fish as sustainable might root in a limited understanding of production practices and their sustainability performance. Whereas low carbon emissions associated with short transportation routes were repeatedly mentioned as a key advantage of Swiss fish products, respondents seem to neglect other energy demands in fish production. The latest aquaculture development projects in Switzerland are mostly farms with indoor recirculating aquaculture systems, which are among the most energy-demanding fish production systems [1, 23]. Energy demand is particularly high when warm-water species such as shrimp or pangasius are farmed. Furthermore, the key inputs of feed and seed are generally imported in all Swiss aquaculture farms, and many species farmed in Switzerland have high feed requirements, with fish-in-fish-out ratios > 1. In other words, while energy use in transporting the final products may be lower for Swiss fish, the overall ecological benefits compared with imported fish might be limited. Assuming that Swiss fish is sustainable per se is also careless from a governance perspective, since aquaculture production is poorly regulated in Switzerland. Nevertheless, local produce benefits from a “subjective bonus”—both by experts and apparently by consumers. Products such as “Swiss shrimp” and “Swiss salmon”, which are not rated sustainable by the WWF, both achieve much higher market prices than imported products labelled sustainable. A similar observation was made by Maesano et al. [18], who observed that worldwide consumers prefer domestic over imported fish products and are willing to pay more for otherwise like-products.

Apart from local produce, fish ecolabels also appear to be instrumentalised for arbitrary market discrimination in Switzerland. Why the WWF, as the apparent dominating actor shaping the sustainable fish agenda, is unconditionally supporting the MSC and ASC labels but not others may be interpreted in different ways. Although the selected labels could indeed represent the only sustainable choices, it is questionable whether the role of the WWF is fully independent from these labels. The WWF was a founding partner of both the MSC and ASC standards, substantially supporting the development and proliferation of both organisations. The observation that the WWF recommends certain MSC-certified fisheries as sustainable even though it initially objected against their certification^19^ suggests a certain bias. Independent and consequent acting would mandate the WWF to withdraw support for the affected fisheries, a measure that only WWF Germany has taken. In contrast, WWF Switzerland justifies its support for the MSC by claiming a lack of alternatives for sustainable fisheries standards. However, a study by Borland and Bailey [4] suggests otherwise. Comparing the MSC with Fair Trade USA (FTUSA) for its suitability with small-scale fisheries, the authors found that FTUSA is not only more inclusive but also achieves better environmental outcomes. Yet the WWF’s seafood guides do not mention FTUSA. A similar situation is evident for aquaculture labels, where the standards GlobalGAP, BAP, and AquaGAP apparently demand very similar requirements for production practices as the WWF-recommended ASC. By only promoting a limited number of fish labels and pushing its retail partners to increase their share of labelled seafood on a yearly basis, the WWF clearly contributes to the elimination of all other fish labels and fish products, whether they are sustainable or not. A similar observation can be made for organic fish labels represented in the Swiss market. Even though WWF Switzerland does not limit organic standards in its seafood guides to specific labels, there appears to be some bias in favour of the organic labels Bio Suisse and Naturland (a German label). Following a recommendation by WWF Switzerland and partner organisations, these labels are considered “superior” to other organic labels such as EU organic or Soil Association Organic (SAO), even though differences are limited.^20^ Two hypotheses may explain this situation. On the one hand, similar to the incorrect assumption that all Swiss fish is sustainable, the preference for the label may stem from a lack of understanding. Rather than based in objectivity, the preferential treatment may be rooted in a preference for Swiss products as such, a perception that could be categorised as “product chauvinism”. The other explanation is the instrumentalisation of a label with the goal to limit unwanted competition. There appears to be a strong link between Coop and Bio Suisse, with evidence that the former has tried to monopolise the Bio Suisse label [21]. Through this lens, labels in Switzerland are not exclusively used to increase the consumption of sustainable products. If that were the case, any label would logically be placed in as many sales channels as possible. Granting exclusivity for a label to specific organisations or denying its use to others follows other objectives and certainly does not serve the cause of a sustainable transformation within the sector. The WWF’s fish consumer guides cannot really be evaluated due to a lack of transparency.

Our findings suggest that the claims, perception, and reality of the “sustainability” of fish products vary significantly. A main reason is that current approaches for differentiating fish products are vague and not transparent, resulting in arbitrary interpretations of sustainability. The resulting “sustainability gap” and an apparent appropriation of the sustainable fish agenda by certain private actors mandates a stronger role for the state to achieve sustainable and fair outcomes.

First, adequate economic incentives would benefit a higher consumption of sustainable products. Price is a key driving force in the RFS sector because of the limited communication potential of sustainable products. This aligns with observations by Giacomarra et al. [13], who found that information is key to consumer decisions concerning fish products. This suggests that trade measures benefitting sustainable products could help increase the share of sustainable consumption where communication of product benefits is challenging. Such a measure would further potentially provide better access for sustainable products that currently face hindrances accessing EU markets [26] or balance unfair economic advantages for unsustainable products [34, 35].

Second, established means for product differentiation fail to be inclusive, a drawback that has already been well covered by academic studies for certification schemes and ecolabels [2, 10, 14, 16, 19, 29, 36].

Third, because private interventions fail to account for diversity. If ecolabels are used as the main vehicle to assess and communicate sustainability, product choice will largely depend on the coverage that these eco-standards offer. Where labelled products are unavailable because the species or production systems are not covered by any eco-standard or no operations have been certified even if sustainable operations exist, affected products will be perceived as unsustainable and might be consumed less or, in the worst case, disappear from the market entirely. A long-term outcome is a limited product range, where offered products are reduced to a handful of main species that benefit from economy of scale. This in turn reduces sustainability from a cultural (and potentially ecological) perspective.

Finally, it is possible to achieve operational frameworks for sustainability across a diverse range of actors with different interests and understanding of sustainability, but only if a common set of criteria is defined and openly shared among stakeholders [27].

We infer from these observations that when using trade measures in the form of product differentiation between sustainable and unsustainable fish products, states must pay utmost attention to their design. The current approaches to differentiate sustainable from unsustainable fish products introduced in Switzerland by private actors not only fail to achieve sustainable outcomes, but they also fall short of meeting all the criteria required for trade-related product differentiation that states may use in WTO conformity. Supporting some labels but not others that achieve similar outcomes would be considered arbitrary and therefore trade obstruction. A preferred treatment for Swiss or local produce on geographic principles would clearly be considered discriminatory. In addition, all approaches appear to be non-inclusive and discriminatory. Closely linked to the latter observation is a current lack of transparency. Without an accordant level of transparency, discrimination cannot be excluded.

Looking forward, a potential option for product differentiation through trade policy brought forward during our interviews is to introduce a state regulation for sustainable fish production, or for sustainable agriculture production more generally, an option that aligns with the proposition of Roheim et al. [29]. Following the example of the Swiss Organic Farming Ordinance or the EU regulations for organic aquaculture,^21^ sustainable production would be defined in the form of a regulation, through which the state would mandate binding requirements for all producers that aim to market their products as sustainable. Such a scenario would have different advantages. First, it would allow sustainability to be defined holistically. Compared with existing schemes, other criteria such as social impacts, transparency, and animal welfare could be added on demand. Second, this more holistic framing of sustainability, openly communicated and verified by the state, would add legitimacy to potential claims and would likely increase consumer support for sustainable products. Third, the regulation could be designed to avoid discrimination across actors and existing standards and labels. Following the examples of the Swiss and EU organic regulations, the standard could explicitly allow the accreditation of third-party schemes that meet all exigencies. Fourth, while regulating imports, state regulation for sustainable production would help bridge existing gaps for a national regulation of aquaculture production in Switzerland. Such a regulation would harmonise requirements for fish production across cantons and guarantee minimal standards of production within the country. Finally, a regulation could be designed to exclude specific production practices when they violate certain principles, without penalising like-products or entire production systems. As an example, the regulation might acknowledge the MSC label as generally sustainable while specific MSC-certified fisheries, which are regarded as violating sustainability principles, could be explicitly excluded. Likewise, all fish production systems involving any forms of human rights violations could also be excluded.

## Conclusion

The present study has looked at how sustainable fish has been defined in Switzerland, what measures the private sector uses to foster the consumption of sustainable fish, and what states could learn from these experiences in terms of fish product differentiation. Feedback from interviewed experts and information obtained through online searches suggest that current differentiation between sustainable and unsustainable fish is not inclusive and that existing tools developed for this purpose are vague and not transparent. Subjective interpretation of sustainability by key actors has apparently led to discrimination in the Swiss fish market, and higher consumption of sustainable fish products is constrained. We have observed that existing measures to differentiate sustainable from unsustainable fish products have shortcomings in terms of WTO compliance and that local production is poorly regulated. Our findings imply that the Swiss state should play a more important role if it aims to fulfil the promise of article 104a of the Swiss Constitution, which seeks to foster sustainable production and cross-border trade relations that contribute towards this goal. We propose that a potential option to increase the production and consumption of sustainable fish is to provide preferred trade treatment for sustainable fish products. When designing measures for product differentiation, a careful choice is paramount so as not to violate existing trade obligations. In addition, “sustainable fish” needs to be defined in a holistic, accessible, and inclusive way. A regulation that defines what sustainable fish production is could potentially fulfil this requirement, but further investigation in the design and content of such a regulation is required. Looking beyond the Swiss border, it can be assumed that our findings are relevant for other countries as well. Many other countries, particularly in Europe, either use the same or very similar means to differentiate sustainable fish products as Switzerland does. Key actors such as the WWF and product differentiation in the form of ecolabels or “consumer guides for fish” play a similar role as they do in Switzerland.^22^ Furthermore, state intervention for claims of sustainable fish products have already been debated in the EU,^23^ and our findings might contribute to the design of appropriate means to advance policy efforts that increase sustainable fish consumption and production in and outside the EU.

### Limitations

The study design and methodologies used have some obvious limitations, such as limited data collection, that were mainly due to capacity constraints. The sample of interviewees might not be representative. In addition, the coronavirus pandemic limited the possibility of face-to-face interviews and resulted in some selected interviewees not having the capacity to participate in our research. It may also have compromised some interviews that needed to be carried out over the phone rather than face-to-face.

In addition, although Switzerland shares many similarities with EU countries, particularly in terms of trade conditions, the Swiss market is relatively small, and specific market requirements and product preferences vary across countries. Transferability of our findings can thus be challenged. Regardless of these shortcomings, we believe that our findings are representative and that conclusions in terms of the research questions would be similar for a larger sample and in other countries that seek to support sustainable fish consumption while complying with WTO law.

## Supplementary Information

**Additional file 1.** Questionnaire used for semi-structured interviews (English translation).

**Additional file 2.** Questionnaire used for semi-structured interviews (original in German).

**Additional file 3.** Questionnaire used for online survey (original in German).

**Additional file 4.** Questionnaire used for online survey (English translation).

**Additional file 5.** Original wording of claims (in German) and corresponding sources for Fig. 3.

## Data Availability

On demand from some interviewees, the authors decided not to publish interview transcripts. This is justified in the fact that the affected industry is small and that certain statements might be linked to the corresponding interviewees, resulting in unwanted consequences or repercussions. The authors are, however, open to consider sharing specific information on the demand, based on a case-to-case decision.

## Abbreviations

ASC: Aquaculture Stewardship Council
FTUSA: Fair Trade USA
MSC: Marine Stewardship Council
RFS: Restaurant and food service
WTO: World Trade Organization
WWF: World Wide Fund

## Footnotes

1 “Fish” is used in this article to cover all fish and seafood destined for human consumption.

2 Under the UN Convention on Biological Diversity.

3 See, for example, Steiner [33].

4 Council Regulation (EC) No. 1005/2008 of 29 September 2008 establishing a Community system to prevent, deter and eliminate illegal, unreported, and unregulated fishing.

5 See, for example, Shaffer [31].

6 The Comprehensive Economic Partnership Agreement between EFTA states and Indonesia based on sustainability provisions for the palm oil sector. See, for example, Sieber-Gasser [32].

7 Questionnaires are available in the “Additional files 1, 2, 3, and 4” section.

8 If the same website was available in more than one language but the content was identical, only one was considered.

9 See WWF [39].

10 See Finding “WWF’s influence on the definition of sustainable fish”.

11 “Restaurant and food service” is used in this article to combine all businesses apart from retail, including restaurants, hotels, delicatessens, and gourmet shops, as well as similar businesses.

12 In Switzerland, these are called “Ratgeber Fische & Meeresfrüchte”. See WWF [40].

13 Although the two categories “recommended” and “recommended labels” may seem the same, they are not.

14 See explanation in the next sub-chapter.

15 North Sea Foundation, Marine Conservation Society, Thünen Institute.

16 As referred to by experts working in the Swiss fish trade sector.

17 All Score 1 and Score 2 products, including the ecolabels ASC, MSC, and organic.

18 Namely, social and economic aspects [27].

19 See WWF [38].

20 See, for example, Bio Suisse [3].

21 Ordinance on Organic Farming and the Labelling of Organically Produced Products and Foodstuffs (Organic Farming Ordinance) of 22 September 1997; EU regulation 710/2009 on the implementation of EU regulation 834/2007 on organic aquaculture animal and seaweed.

22 The WWF publishes its “consumer guide for fish” in 25 other countries, apart from Switzerland, and has partnerships with major retailers in other markets as well.

23 See EC [12].
