# Supplementary material for: Drawing the line between sustainable and unsustainable fish: product differentiation that supports sustainable development through trade measures
Source: Environ Sci Eur. 2021 Sep 30;33(1):113. doi: 10.1186/s12302-021-00551-6 (PMC8481322; doi:10.1186/s12302-021-00551-6)
Supplement: Supplementary file 1 — Additional file 1. Questionnaire used for semi-structured interviews (English translation). [file 12302_2021_551_MOESM1_ESM.pdf]

Short presentation / introduction

- *Brief introduction to person, research project (goals, client, partner) and study (goal, format, expectations, timelines).*
- *Explanation of privacy protection and voluntary participation. Explain / offer the possibility of terminating the interview at any time.*
- *Get verbal consent for participation.*
- *Questions about the procedure?*

Guiding questions

Sustainable fish: definition, implementation, actors

1. What means 'sustainable fish' for you?
2. Who defines what sustainable fish is in Switzerland?
    a. Can you provide more details: e.g. how long have these actors been active?
    b. How big is their influence? E.g. on other actors?
3. To what extent are their respective definitions implemented?
    a. By which means?
    b. To which extent?
4. How do you rate that in terms of sustainability?

Labels

5. Do you think that the following labels are sustainable? If not, why not?
    a. organic
    i. -> are there differences across organic labels (e.g. EU organic, Naturland, New Zealand organic, Alnatura)?
    b. MSC
    c. ASC
    d. GlobalGAP
    e. FOS, AquaGAP, FairTrade, BAP (GAA)
6. Are there other labels that you consider sustainable? If yes, which?

Swiss / local fish

7. How do you rate Swiss fish in terms of sustainability?
8. What basis do you use for this assessment?
9. Are there differences between farmed and wild-caught products?
10. What is the legal framework for fisheries and aquaculture in Switzerland?
11. Who are the key players in the implementation / control of fish sustainability standards in Switzerland?
12. Are there differences across cantons in legislation and / or implementation?

Market / market shares

13. Into which sub-markets/sectors can the Swiss fish market be divided?
14. Can you give me an indication of the respective share of these 'submarkets' in the total turnover (in% of the total fish market, estimated)?
15. Do you know any official bodies that collect data on market shares?
16. Can you indicate me the respective actors in the sub-markets?
17. How high do you estimate the respective shares of sustainable products in these sub-markets (%)?
    a. Do these indications correspond to your personal definition of sustainability?
    b. If not, what is the definition of sustainability that you use as a basis?
    c. Are there any differences between the two?
    d. Are there reliable sources / data to answer all these questions?

Future / potential intervention or regulations in the market by the state

18. What do you think about the proposition that in the future only sustainable fish should be sold in Switzerland (and as a result, for example, some species or products from certain fisheries that are not sustainable, would no longer be available)?
19. What do you think about the idea that in the future only the 'WWF recommended labels ASC, MSC and organic labels' should be allowed to be marketed in Switzerland (and e.g. certain species/products would no longer be available)?
20. Do you see other ways of defining 'sustainable fish' for such a purpose?
21. If there shall only be sustainable fish in the future, who do you think should define what 'sustainable fish' is?
22. And who should monitor / ensure the implementation?

Other potential interviewees and sources of information

23. Do you know important sources of information that can provide data and answers to these questions?
24. Can you give me the names of people and organizations that you consider relevant to this study / survey?

Closing questions and personal information

25. Are there any questions / concerns that you would like to share?
26. In which sector and working field are you active?
27. What is your expertise and focus on fish and the research topic (e.g. sustainability, markets and trade, quality, other)?
28. What is your current job title / title?

- *Thanks.*
- *Repeat once more that participation is voluntarily and can be revoked retrospectively.*
- *Explain when research results can be expected, the further course of the research project (and the opportunity to find out more about it) and the intention of research team to share the results of the study as soon as they have been evaluated / published.*
