# Supplementary material for: Drawing the line between sustainable and unsustainable fish: product differentiation that supports sustainable development through trade measures
Source: Environ Sci Eur. 2021 Sep 30;33(1):113. doi: 10.1186/s12302-021-00551-6 (PMC8481322; doi:10.1186/s12302-021-00551-6)
Supplement: Supplementary file 2 — Additional file 2. Questionnaire used for semi-structured interviews (original in German). [file 12302_2021_551_MOESM2_ESM.pdf]

Kurze Vorstellung / Einführung

- *Kurze Einführung zu Person, Forschungsprojekt (Ziele, Auftraggeber, Partner) und Studie (Ziel, Format, Erwartungen, Timelines).*
- *Erläuterung Datenschutz und Freiwilligkeit der Partizipation. Möglichkeit zum jederzeitigen Abbruch des Interviews erläutern/anbieten.*
- *Mündliche Einwilligung (‚Consent‘) für Partizipation abholen.*
- *Fragen zum Vorgehen?*

Fragen

Nachhaltiger Fisch: Definition, Umsetzung, Akteure

1. Was ist für Sie ‚nachhaltiger Fisch‘?
2. Wer definiert in der Schweiz, was nachhaltiger Fisch ist?
    a. Können Sie mehr Details dazu geben: z.B. wie lange sind diese Akteure bereits aktiv?
    b. Wie gross ist deren Einfluss auf welche andere Akteure?
3. Inwiefern wird deren jeweilige Definition umgesetzt?
    a. Durch welche Mittel?
    b. In welchem Umfang?
4. Wie schätzen Sie das ein in Bezug auf Nachhaltigkeit?

Labels

5. Glauben Sie, dass die folgenden Labels nachhaltig sind? Falls nein, wieso nicht?
    a. Bio
        i. -> gibt es Unterschiede zwischen den Bio-Labels (z.B. EU Bio, Naturland, Neuseeland Bio, Alnatura)?
    b. MSC
    c. ASC

    d. GlobalGAP
    e. FOS, AquaGAP, FairTrade, BAP (GAA)
6. Gibt es andere Label, die Sie als nachhaltig betrachten? Wenn ja, welche?

Schweizer / lokaler Fisch

7. Wie beurteilen Sie Schweizer Fisch bezüglich Nachhaltigkeit?
8. Welche Beurteilungsgrundlage nutzen Sie für diese Einschätzung?
9. Gibt es Unterschiede zwischen Zucht und Wildfang Produkten?
10. Wie sieht der gesetzliche Rahmen zu Fischerei und Aquakultur aus?
11. Wer sind wichtige Akteure in der Umsetzung/Kontrolle von Nachhaltigkeitsstandards Fisch in der Schweiz?
12. Gibt es kantonale Unterschiede in der Gesetzgebung und/oder Umsetzung?

Markt / Marktanteile

13. In welche Teilmärkte kann der Schweizer Fisch-Markt eingeteilt werden?
14. Können Sie mir eine Angabe zum jeweiligen Anteil im Gesamtumsatz der Teilmärkte geben (in % von totalem Fischmarkt, geschätzt)?
15. Kennen Sie dazu Statistiken / offizielle Stellen, welche Daten zu Marktanteilen erheben?
16. Können Sie mir die jeweiligen Akteure in den Teilmärkten angeben?
17. Wie hoch schätzen Sie die jeweiligen Anteile an nachhaltigen Produkten in diesen Teilmärkten (%)?
    a. Entsprechen diese Anteile ihrer persönlichen Definition von Nachhaltigkeit?
    b. Wenn nicht, entsprechend welcher Definition?
    c. Gibt es Unterschiede?
    d. Gibt es dazu verlässliche Quellen/Daten?

Zukunft / mögliche Interventionen / Regulierung des Marktes durch den Staat

18. Was hielten Sie davon, wenn es in Zukunft in der ganzen Schweiz nur noch nachhaltigen Fisch geben würde (und z.B. als Folge manche Spezies oder Produkte aus gewissen Fischereien nicht mehr erhältlich sind)?

19. Was hielten Sie davon, wenn es in Zukunft nur noch die vom WWF empfohlenen Labels ASC, MSC und Bio gibt (und z.B. gewisse Spezies nicht mehr erhältlich sind)?

20. Sehen Sie andere Möglichkeiten, um ‚Nachhaltigen Fisch' in einem solchen Kontext zu definieren?

21. Wenn es in Zukunft nur nachhaltigen Fisch geben soll, wer sollte Ihrer Meinung nach definieren, was ‚nachhaltiger Fisch' ist?

22. Und wer sollte die Umsetzung überwachen / sicherstellen?

Nachhaltigkeitsexperten / Informationsquellen

23. Kennen Sie wichtige Informationsquellen, welche Daten und Antworten zu diesen Fragen liefern können?

24. Können Sie mir Namen von Personen und Organisationen angeben, welche Sie für diese Studie/Umfrage als relevant erachten?

Abschliessende Fragen und persönliche Angaben

25. Gibt es aus Ihrer Sicht Fragen / Anliegen, die Sie gerne teilen würden?

26. In welchem Arbeitsfeld sind Sie tätig?

27. Was sind Ihre Expertise und Schwerpunkt in Bezug zu Fisch und dem Forschungsthema (z.B. Nachhaltigkeit, Märkte und Handel, Qualität, anderes)?

28. Wie lautet Ihr/e momentane/r Stellenbezeichnung/Titel?

- *Danke.*
- *Nochmals wiederholen, dass Partizipation freiwillig und auch im Nachhinein widerrufen werden kann.*

- *Erläutern, wann mit Forschungsergebnissen zu rechnen ist, weiterer Verlauf des Forschungsprojekts (und Möglichkeit sich darüber zu informieren) und Absicht, Ergebnisse der Teilstudie zukommen zu lassen, sobald diese ausgewertet/publiziert.*
