# Supplementary material for: Drawing the line between sustainable and unsustainable fish: product differentiation that supports sustainable development through trade measures
Source: Environ Sci Eur. 2021 Sep 30;33(1):113. doi: 10.1186/s12302-021-00551-6 (PMC8481322; doi:10.1186/s12302-021-00551-6)
Supplement: Supplementary file 3 — Additional file 3. Questionnaire used for online survey (original in German). [file 12302_2021_551_MOESM3_ESM.pdf]

Nachhaltigkeit von Fischprodukten im Schweizer Markt

1. Was ist für Sie ‚nachhaltiger Fisch'?

2. Wer definiert in der Schweiz, was 'nachhaltiger Fisch' ist?

3. Mit welchen Mittel wird diese Definition (resp. die Interpretation von 'nachhaltigem Fisch') umgesetzt?

4. Inwiefern (in welchem Umfang) gelingt diese Umsetzung?

5. Wie schätzen Sie sowohl Definition wie Umsetzung in Bezug auf Nachhaltigkeit ein (Fragen 2 bis 4)?

6. Glauben Sie, dass die folgenden Labels nachhaltig sind (es folgt eine Auswahl)?

MSC
Ja
Nein -> 7. Wieso nicht

8. ASC
Ja
Nein -> 9. Wieso nicht

10. Bio
Ja
Nein -> 11. Wieso nicht

12. Sind alle Bio-Labels (z.B. Bio Suisse, EU Bio, Naturland) gleich bezüglich Nachhaltigkeit oder gibt es Unterschiede?

13. Gibt es andere Label für Fisch, welche Sie als nachhaltig betrachten? Wenn ja, welche?

14. Wie beurteilen Sie Schweizer Fisch bezüglich Nachhaltigkeit?

15. Was hielten Sie davon, wenn es in Zukunft in der ganzen Schweiz nur noch

nachhaltigen Fisch geben würde (und z.B. gewisse Spezies dann nicht mehr erhältlich sind)?

16. Was hielten Sie davon, wenn es in Zukunft nur noch die Labels ASC, MSC und Bio gibt (und andere Produkte oder gewisse Spezies nicht mehr erhältlich sind)?

17. Sehen Sie andere Möglichkeiten (als die bisher genannten), um ‚Nachhaltigen Fisch' zu definieren?

18. Wenn es in Zukunft nur nachhaltigen Fisch geben sollte, wer müsste Ihrer Meinung nach definieren, was ‚nachhaltiger Fisch' ist?

19. Und wer sollte die Umsetzung überwachen / sicherstellen?

Angaben zu ihrer Person (nicht zwingend)

Die von Ihnen gemachten Angaben werden alle streng vertraulich behandelt und nur in anonymisierter Form weiterverwendet.
Persönliche Angaben werden nur für statistische Zwecke (z.B. gibt es Unterschiede in den Antworten je nach Position oder Altersgruppe?) oder für allfällige Rückfragen verwendet. Diese Angaben sind nicht zwingend auszufüllen und können auch übersprungen werden.
Wir danken Ihnen vielmals, dass Sie sich die Zeit genommen haben, diesen Fragebogen zu beantworten.

20. Name und Vorname

21. Firma

22. Position/Rolle

23. Altersgruppe

jünger als 30
zwischen 30 und 45
45 oder älter
