# Supplementary material for: Drawing the line between sustainable and unsustainable fish: product differentiation that supports sustainable development through trade measures
Source: Environ Sci Eur. 2021 Sep 30;33(1):113. doi: 10.1186/s12302-021-00551-6 (PMC8481322; doi:10.1186/s12302-021-00551-6)
Supplement: Supplementary file 4 — Additional file 4. Questionnaire used for online survey (English translation). [file 12302_2021_551_MOESM4_ESM.pdf]

Sustainability of fish products in the Swiss market

1. What means 'sustainable fish' for you?

2. Who in Switzerland defines what 'sustainable fish' is?

3. How is this definition (or their interpretation of 'sustainable fish') implemented?

4. In how far (to what extent) is this implementation successful?

5. How do you rate both definition and implementation with regard to sustainability (questions 2 to 4)?

6. Do you think the following labels are sustainable (a selection follows)?

MSC
Yes
No -> 7. Why not?

8. ASC
Yes
No -> 9. Why not?

10. Bio
Yes
No -> 11. Why not?

12. Are all organic labels (e.g. Bio Suisse, EU Bio, Naturland) the same in terms of sustainability or are there differences?

13. Are there other labels for fish that you consider sustainable? If yes, which?

14. How do you rate Swiss fish in terms of sustainability?

15. What do you think of the idea that in the future only sustainable fish should be allowed to be sold in Switzerland (and consequently certain species or products would no longer be available)?

16. What do you think of the idea that for this purpose in the future only the labels ASC, MSC and organic labels should be sold (and other products or certain species would no longer be available)?

17. Do you see other ways (than those mentioned so far) to define 'sustainable fish'?

18. If only sustainable fish should be sold in the future, who do you think should define what 'sustainable fish' is?

19. And who should monitor / ensure its implementation?

Personal information (not mandatory)

The information you provide will be treated with strict confidentiality and will only be used in anonymised form.
Personal information is only used for statistical purposes (e.g. are there differences in the answers depending on the position or age group?) or other queries. Personal information does not have to be filled out and can also be skipped.
Thank you very much for taking your time to answer this questionnaire.

20. Surname and First Name

21. Organisation/Company

22. Position/Role

23. Your age
Younger than 30
Between 30 and 45
45 or older
