# Supplementary material for: Drawing the line between sustainable and unsustainable fish: product differentiation that supports sustainable development through trade measures
Source: Environ Sci Eur. 2021 Sep 30;33(1):113. doi: 10.1186/s12302-021-00551-6 (PMC8481322; doi:10.1186/s12302-021-00551-6)
Supplement: Supplementary file 5 — Additional file 5. Original wording of claims (in German) and corresponding sources for Fig. 3. [file 12302_2021_551_MOESM5_ESM.pdf]

Supporting material for Figure 3: original wording of claims and corresponding sources

Company	Claim in original language (German)	Source(s):
SM1	"aus nachhaltigen Quellen, wie sämtlicher Fisch in der Migros", "100 % nachhaltiger Fisch"	Migros (2017). Generation M: Versprechen nachhaltiger Fisch. https://www.youtube.com/watch?v=6gSauLroysU. Accessed 24 August 2021.<br>Migros (2018). Migros Sommer: Grillitarier – Fisch. https://www.youtube.com/watch?v=D6O3WatT-aQ. Accessed 24 August 2021.
SM2	"100 % nachhaltig: frisch und tiefgekühlt.<br>Wer sich für den Erhalt der Bestände und der Artenvielfalt in den Meeren einsetzen will, darf keine Kompromisse eingehen. Bei Coop sieht das so aus: 100 %unserer Fische und Meeresfrüchte stammen aus nachhaltigen Quellen, ob frisch, tiefgekühlt, in Dosen oder im Restaurant"	Coop (2016). Taten statt Worte. Bei uns ist Fischgenuss nachhaltig. https://www.coop.ch/content/dam/act/TatenstattWorte_Relaunch/Nachhaltigkeitsthemen/Tierwohl/Nachhaltiger-Fisch/infobroschuere-taten-statt-worte-fisch-2016_de.pdf. Accessed 24 August 2021.
SM3	"100 % unserer dauerhaft verfügbaren Fischprodukte und Meeresfrüchte sind MSC, ASC oder Bio zertifiziert."	Lidl (2021). Nachhaltiges Fischsortiment. Massnahme #192. https://gesagt-getan.lidl.ch/de/detail/Nachhaltiges-Fischsortiment. Accessed 24 August 2021.
SM4	"Seit 2018 verkauft Denner nur noch Fisch und Meeresfrüchte aus nachhaltigen Quellen"	Denner (2021). Denner Richtlinien. https://www.denner.ch/de/qualitaet/qualitaetsanspruch/uebersicht/lebensmittel/. Accessed 24 August 2021.
